# ADAMTS-13-regulated nuclear factor E2-related factor 2 signaling inhibits ferroptosis to ameliorate cisplatin-induced acute kidney injuy: **Running title**: Role of ADAMTS-13 and ferroptosis in AKI.

**DOI:** 10.1080/21655979.2021.1994707

**Published:** 2021-12-07

**Authors:** Xiaoyan Meng, Wenjing Huang, Weiwei Mo, Tingting Shu, Haoqiang Yang, Haibo Ning

**Affiliations:** a Department of Nephrology, The Fourth Affiliated Hospital of Guangxi Medical University, Liuzhou, China; b Department of General Surgery, The Fourth Affiliated Hospital of Guangxi Medical University, Liuzhou, China

**Keywords:** ADAMTS-13, ferroptosis, acute kidney injury, cisplatin, Nrf2 signaling

## Abstract

ADAMTS-13 plays an important role in acute kidney injury (AKI), but the mechanism of cisplatin (CP) induced AKI remains unclear. Ferroptosis is increased in CP-induced AKI, and ADAMTS13 levels are associated with ferritin expression. In this article, we will explore the relationship between the three. After CP induction, mice were given 0.1 and 0.3 nmol/kg ADAMTS-13, and then serum creatinine (Scr) and blood urea nitrogen (BUN) were detected by the kits. The pathological changes of renal tissue were observed by staining with HE and PAS staining, and Western blot detected the expressions of KIM1 and NGAL in renal tissu. Perl’s staining detected iron deposition in renal tissues, the kits detected iron levels, and western blot detected the expression of ferroptosis related proteins. Then the mechanism was further explored by adding ferroptosis inhibitors Ferrostatin 1 (Fer-1) and iron supplements Fe. The expression of Nrf2 pathway related proteins were detected by Western blot. We found that ADAMTS13 alleviated CP-induced ferroptosis in AKI mice with renal function impairment and tubular damage. Fer-1partially reversed CP-induced AKI, and Fe exacerbated this effect. ADAMTS13 alleviated CP-induced inflammatory response and oxidative stress in AKI mice, during which the Nrf2 signaling pathway was abnormal. Overall, ADAMTS-13-regulated Nrf2 signaling inhibits ferroptosis to ameliorate CP-induced AKI.

## Introduction

### Background

Acute kidney injury (AKI) is a common and increasingly frequent condition that generates risks of adverse events and high costs [1].It is characterized by a sharp decline in renal function, nephrotoxic drugs, ischemia reperfusion, and sepsis can lead to AKI [2]. Cisplatin (CP) plays an important role in the treatment of various cancers, but CP can accumulate in proximal renal tubule cells, causing severe nephrotoxicity [3,4]. At present, the mechanism of AKI induced by CP is still not fully understood.

ADAMTS-13 (a disintegrin and metalloprotease with thrombospondin type I domain 13) is a metalloprotease responsible for cleavage of ultra-large von Willebrand factor (VWF) multimers [5]. It was originally identified in the study of thrombotic thrombocytopenic purpura [6]. Studies have shown that recombinant ADAMTS-13 (rh ADAMTS-13) can be used for the treatment of patients with thrombotic thrombocytopenic purpura, reducing AKI in patients [7,8]. Hereditable ADAMTS-13 deficiency is manifested as recurrent AKI [9]. In addition, ADAMTS-13 protects mice from renal ischemia-reperfusion injury by reducing inflammation and improving endothelial function [10].Therefore, whether ADAMTS-13 can be used in the treatment of AKI caused by other causes has attracted our attention, and the specific mechanism of the treatment needs to be further studied.

Ferroptosis is a specific cell death, which is different from cell apoptosis, necrosis, pyrotosis and autophagy. It is characterized by intracellular iron overload and lipid peroxidation caused by reactive oxygen species (ROS) accumulation [11]. Studies have shown that high serum iron levels are associated with increased short-term and long-term mortality in ICU patients with AKI [12]. Ferroptosis inhibitors significantly reduce blood urea nitrogen (BUN), serum creatinine (Scr), and tissue damage in CP-induced AKI [13]. Some drugs that scavenge lipid peroxyl radicals can help control ferroptosis-related disorders, including AKI [14]. Therefore, inhibition of ferroptosis is an effective treatment for CP induced AKI.

Studies have shown that ADAMTS-13 can inhibit oxidative stress and improve the progression of chronic kidney disease after ischemia/reperfusion injury, while rhADAMTS-13 can inhibit ROS level, improve microvascular dysfunction, inhibit GSK3β activity and upregulate the expression of Nrf2 [15]. Nrf2 is the basis and inducible expression of many genes encoding detoxifying enzymes, antioxidant proteins, allobiotic transporters and other stress response mediators [16]. Nrf2 plays an important role in AKI. Regulating the expression of Nrf2/HO-1 and Bcl-2 can reduce CP induced AKI [17]. In addition, study has shown that dimethyl fumarate can prevent ferroptosis and reduce AKI by acting on Nrf2 [18]. The level of Nrf2 has been directly correlated with ferroptosis sensitivity, as increased expression of Nrf2 prevents ferroptosis, whereas decreased Nrf2 enhances the sensitivity of cancer cells to pro-ferroptotic agents [19,20]. Moreover, in children with β-thalassemia, ADAMTS-13 level was positively correlated with platelet count, and negatively correlated with serum ferritin [21]. However, the relationship between ADAMTS-13, Nrf2, and ferroptosis in CP-induced AKI has not been reported so far.

Therefore, it is reasonable to assume that ADAMTS13 may regulate Nrf2 expression and ferroptosis in CP-induced AKI and this study aims to explore the mechanism of ADAMTS-13 in CP induced AKI and its regulation mechanism on ferroptosis, thus providing a theoretical basis for the treatment of CP induced AKI.

## Material and methods

### AKI model construction and treatments

C57BL/6 mice (8–12 week) mice purchased from Cavens (Changzhou, China) were divided into four groups: control (saline), CDDP only (CP, 20 mg/kg; MCE, United States), CP (20 mg/kg) + ADAMTS13 (0.1 nmol/kg) and CP (20 mg/kg) + ADAMTS13 (0.3 nmol/kg). Each group contained 5 mice. 0.1 and 0.3 nmol/kg rhADAMTS13 were injected into the caudal vein daily for 3 days after surgery. All animal experiments comply with the ethical requirements of the animal council. The experiment was approved by the Ethics Committee of The Fourth Affiliated Hospital of Guangxi Medical University. To further examine the role of ferroptosis in it. Mice were randomly divided into five groups by ferroptosis inhibitor Fer-1 and iron supplement ferric citrate (Fe): Control group, CP group, CP (20 mg/kg) + ADAMTS13 (0.3 nmol/kg) group, Fer-1 + CP + ADAMTS13 group and Fe + CP + ADAMTS13 group. The Fer-1 + CP + ADAMTS13 group was injected with 1.5 mg/kg Fer-1 (HY-100579, MCE, United States) through caudal vein before injection of CP, and the Fe + CP + ADAMTS13 group was injected with 15 mg/kg ferric citrate (HY-B1645, MCE, United States) through caudal vein before injection of CP [15,22]. CP was purchased from MCE. Recombinant human ADAMTS13 (rhADAMTS13) were purchased from R&D Systems (Minneapolis, MN).

### Measurement of BUN and Scr

BUN (C013-2, Nanjing Jiancheng Bioengineering Institute, China) and Scr (ab65340, Abcam) levels in serum were measured using the corresponding detection kits in accordance with the manufacturer’s instructions.

### Histopathological analysis

After the mice were sacrificed, part of the kidney tissues was taken and fixed with 10% formaldehyde quickly, dehydrated, embedded into paraffin sections, and stained with H&E. The pathological morphology of the kidney tissues was observed under light microscope [23]. HE staining was used to detect the pathological status of the whole renal tissue, including injury of renal tissue. Renal tissue was damaged, renal tubular epithelial cells were necrotic and exfoliated, lumen was deformed and vacuolized; the glomerulus atrophied, the space became larger, the tissue structure was loose and irregular (The green arrow marks the figure).

### PAS staining

The kidney was embedded in paraffin block with 4 µm sections. Paraffin sections were deparaffinised and rehydrated using serial xylene and alcohol. Specimens were then stained with Periodic Acid-Schiff (PAS) to determine tubular injury [24].

### Western blot

0.1 g of renal tissue was added with 1 mL of RIPA lysate (Beyotime Institute of Biotechnology) and placed on ice, and homogenized thoroughly by a homogenizer. The protein concentration was then detected using a BCA kit (Bio-Rad Laboratories, Inc.). The denatured proteins (30 μg) were separated by SDS-PAGE gel electrophoresis. The isolated proteins were transferred to PVDF membrane and sealed with 5% skimmed milk powder for 2 h. The proteins were incubated overnight in primary antibody at 4 °C. On the second day, PVDF membrane was incubated in the secondary antibody for 2 h and developed with enhanced chemiluminescence kit(GE Healthcare) and Image J software (version 146; National Institutes of Health, Bethesda, MD, USA) was used to analyze the fold-changes of protein levels. The primary antibody information is as follows:anti- KIM1 (ab66062, Abcam, UK), anti-NGAL (ab23477, Abcam, UK), anti- SLC7A11 (ab175186, Abcam, UK), anti- GPX4 (ab125006, Abcam, UK), anti- FTH1 (ab75972, Abcam, UK), anti- FPN1 (ab78066, Abcam, UK), anti- Nrf2 (ab62352, Abcam, UK), anti- HO-1 (ab68477, Abcam, UK), anti- Lamin B (ab232731, Abcam, UK), anti-GAPDH (ab8245, Abcam, UK).

### Perl’s staining

The iron content in tissue sections was determined using an iron stain kit (ab150674; Cambridge, UK). Histological sections were deparaffinized and rehydrated. Slides containing tissue sections were incubated in the Perl’s solution(5% potassium ferrocyanide and hydrochloric acid solutions) for 3 min, washed with distilled water, stained using 3,3‐diaminobenzidine (DAB; Vector Laboratories, Burlingame, USA), and hematoxylin (Sigma‐Aldrich, St Louis, MO, USA) for 5 min, and then washed with distilled water. Finally, the sections were dehydrated in 95% ethanol, followed by absolute ethanol. Three sections per animal were viewed and photographed under a microscope (Nikon TE300; Nikon) [25].

### Iron concentration detection

To detect iron concentration in rental tissue, an iron assay kit (MAK025, Sigma-Aldrich) was used according to the manufacturer’s instructions.

### Fe^2+^ assay

To detect Fe^2*+*^ content in the cells, an iron assay kit (ab83366, Abcam, UK) and presented as nanogram Fe^2*+*^ per milligram of protein according to the manufacturer’s instructions.

### ELISA

TNF-α (H052), IL-1β (H002), IL-6 (H007-1-1) and MPO (A044-1-1) levels in renal tissues were measured using the corresponding ELISA kits purchased form Nanjing Jiancheng Bioengineering Institute in accordance with the manufacturer’s instructions.

### Measurement of oxidative stress levels

SOD (A001-3), GSH (A006-2), MDA (A003-1) and ROS (E004) levels in renal tissues were measured using the corresponding detection kits purchased form Nanjing Jiancheng Bioengineering Institute in accordance with the manufacturer’s instructions.

### Statistical analysis

The data are presented as the mean ± standard deviation. All experiments were performed in triplicate. Statistical significance in the study was analyzed by one-way ANOVA followed by Tukey’s post hoc test and Bonferroni’s correction. *P* < 0.05 was considered to indicate a statistically significant difference. The analyses were performed using SPSS 17.0 software (SPSS, Inc.).

## Results

### rhADAMTS13 alleviated renal function damage in CP-induced AKI mice

**Figure 1.**
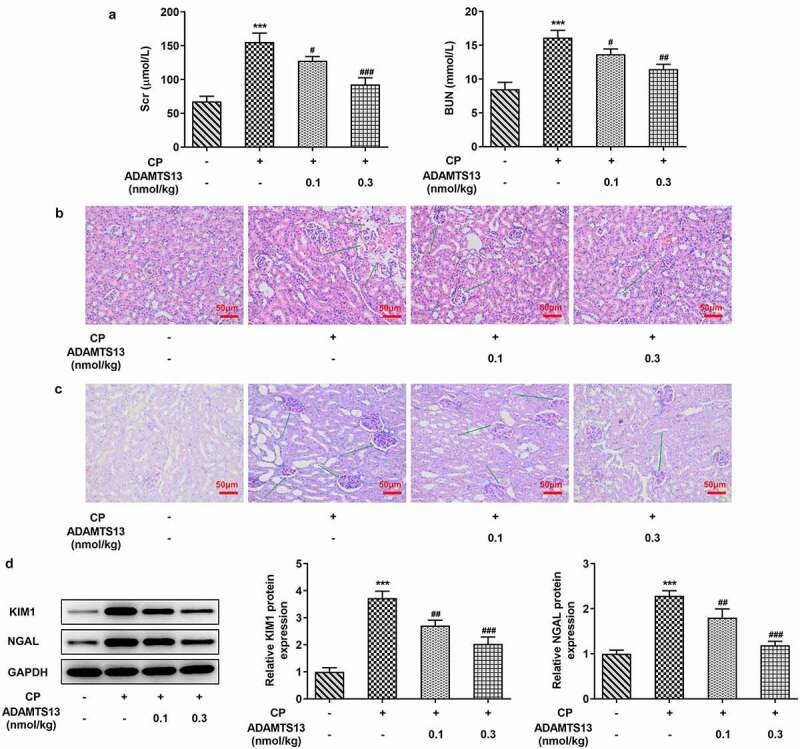
rhADAMTS13 alleviated renal function damage in CP-induced AKI mice. (a). Serum levels of renal function indexes Scr and BUN were detected by the kits. n = 5. The histopathological changes of kidney were observed by HE (b) and PAS staining (c). n = 3. magnification×200.(d). Western blot detected the expression of KIM1 and NGAL. ***P < 0.001 vs control. #P < 0.05, ##P < 0.01, ###P < 0.001 vs CP

**Figure 2.**
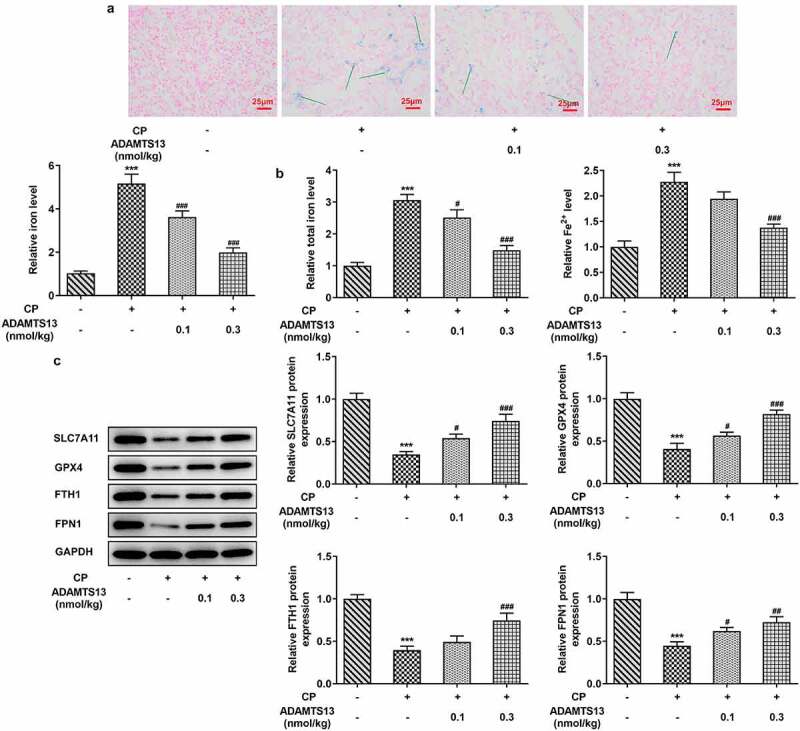
rhADAMTS13 alleviated ferroptosis in CP-induced AKI mice. (a). Perl’s staining was used to detect iron deposition in renal tissues. n = 3. (b). The kits detect total molten iron and Fe^2+^. magnification×200. n = 5. (c). Western blot were used to detect the expression of ferroptosis related proteins. n = 3. ***P < 0.001 vs control. #P < 0.05, ###P < 0.001 vs CP

In order to detect the effect of rhADAMTS13 on CP-induced AKI in mice, we used the kit to detect the concentrations of BUN and Scr in serum of mice. We found that the concentrations of BUN and Scr in mice were significantly increased after CP induction compared with the control group. The concentrations of BUN and Scr in 0.1 nmol/kg ADAMTS13 and 0.3 nmol/kg ADAMTS13 groups decreased in a dose-dependent manner (Figure 1a). Subsequently, HE and PAS staining were used to observe the histopathological changes of kidney in mice. Renal tubules were obviously damaged in CP induced AKI mice, and the epithelial cells of renal tubules were necrotic and exfoliated, and the lumen was deformed and vacuolated. And the glomerulus atrophied, the space became larger, the tissue structure was loose and irregular. 0.1 nmol/kg ADAMTS13 and 0.3 nmol/kg ADAMTS13 significantly reduced renal injury, especially in the high-dose group (Figure 2b and c).Western blot was used to detect the expression of KIM1 and NGAL proteins associated with renal tissue injury. We found that the expression of KIM1 and NGAL in CP group was significantly increased compared with the control group. While administration of 0.1 nmol/kg and 0.3 nmol/kg ADAMTS13 reversed this phenomenon (Figure 1c). The results showed that rhADAMTS13 could alleviate renal function damage in CP-induced AKI mice.

### rhADAMTS13 alleviated ferroptosis in CP-induced AKI mice

In order to detect the expression of ferroptosis related indicators in AKI mice after rhADAMTS13 treatment, we used Perl’s staining to detect iron deposition in renal tissues. The results showed that iron deposition in renal tissues was significant after CP induction (The blue ones are iron deposits), while iron deposition in renal tissues after administration of 0.1 nmol/kg ADAMTS13 and 0.3 nmol/kg ADAMTS13 decreased in a dose-dependent manner (Figure 2a).Subsequently, we used relevant kits to detect the expression of total iron and Fe^2+^ in renal tissues, and the results showed that compared with the control group, the expression of total iron and Fe^2+^ in CP group increased significantly. Compared with CP group, the expression of total iron and Fe^2+^ in CP+ADAMTS13 (0.1 nmol/kg) and CP+ADAMTS13 (0.3 nmol/kg) groups were down-regulated (Figure 2b).The expressions of ferroptosis related proteins SLC7A11, GPX4, FTH1 and FPN1 were detected by Western blot. We found that compared with the control group, the expressions of SLC7A11, GPX4, FTH1 and FPN1 in CP group were significantly decreased, indicating that ferroptosis in kidney tissue in AKI mice induced by CP was enhanced. Compared with CP group, SLC7A11, GPX4, FTH1 and FPN1 expression in CP+ADAMTS13 (0.1 nmol/kg) and CP+ADAMTS13 (0.3 nmol/kg) groups were reversed. It was shown that rhADAMTS13 alleviated CP-induced ferroptosis in AKI mice (Figure 2c).

### Fer-1 partially reversed CP induced AKI, while Fe increased AKI

**Figure 3.**
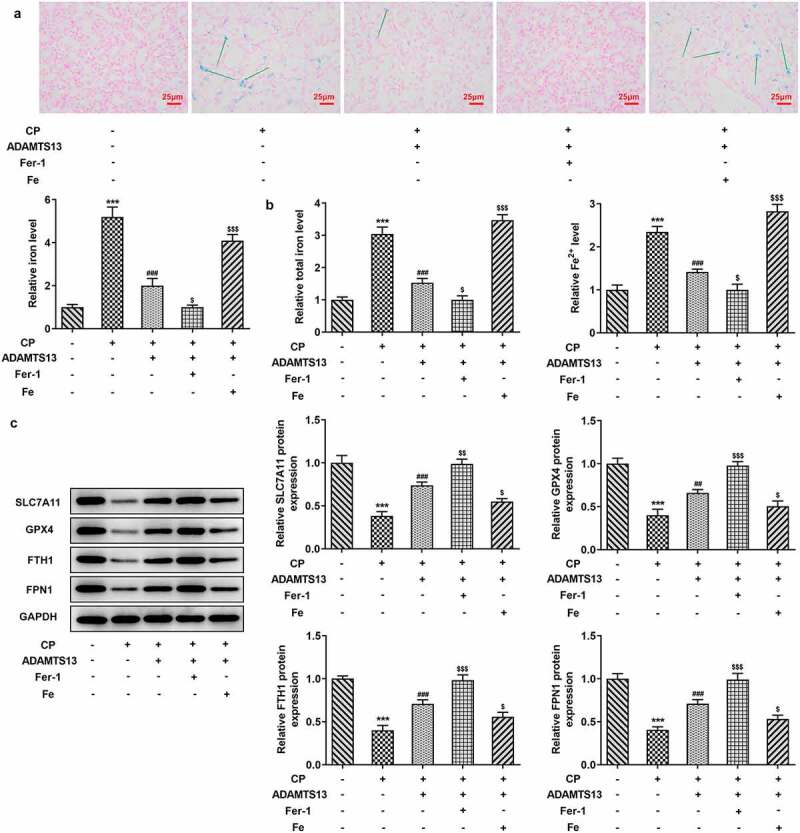
The expression of iron in the renal tissues of Fer-1 and Fe was given. (a). Perl’s staining was used to detect iron deposition in renal tissues after given with Fer-1 or Fe. n = 3. (b). The kits detect total molten iron and Fe^2+^ after given with Fer-1 or Fe. n = 5. (c). Western blot were used to detect the expression of ferroptosis related proteins after given with Fer-1 or Fe. n = 3. ***P < 0.001 vs control. ##P < 0.01, ###P < 0.001 vs CP. $p < 0.05, $$p < 0.01, $$$p < 0.001 vs CP + ADAMTS13

**Figure 4.**
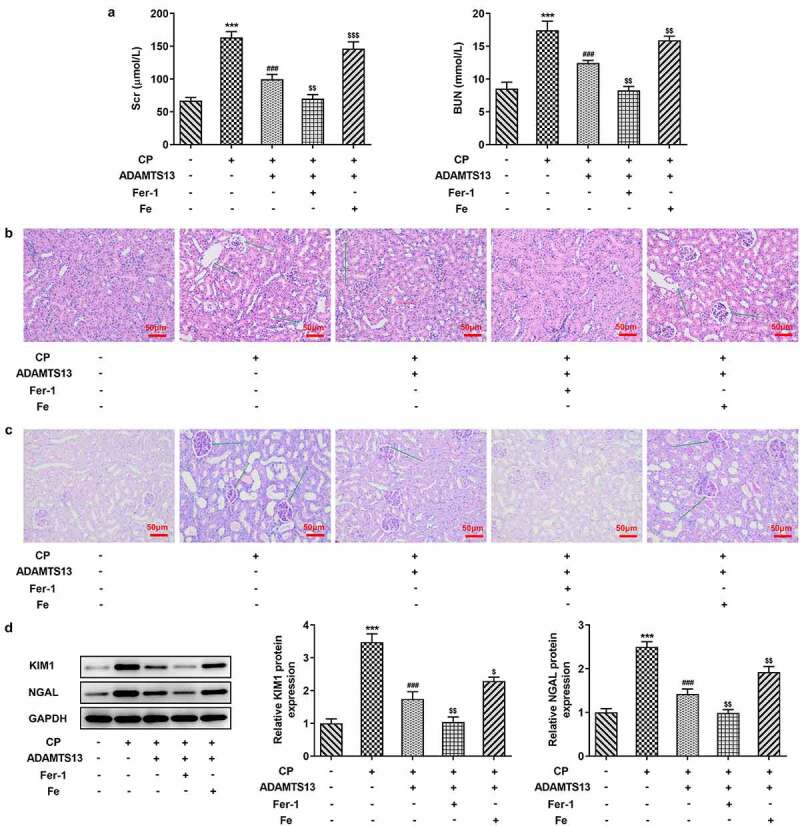
Fer-1 partially reversed CP induced AKI, while Fe increased AKI. (a). Serum levels of renal function indexes Scr and BUN were detected by the kits after given with Fer-1 or Fe. n = 5. The histopathological changes of kidney were observed by HE (b) and PAS staining (c) after given with Fer-1 or Fe. n = 3.(d). Western blot detected the expression of KIM1 and NGAL after given with Fer-1 or Fe. n = 3. ***P < 0.001 vs control. ###P < 0.001 vs CP. $p < 0.05, $$p < 0.01, $$$p < 0.001 vs CP + ADAMTS13

We further explored the mechanism by adding ferroptosis inhibitor Fer-1 and iron supplement Fe. In addition, 0.3 nmol/kg ADAMTS13 was selected for the experiment. Perl’s staining results showed that compared with CP+ADAMTS13 group, Fer-1 could further inhibit iron deposition in renal tissue, while Fe reversed the inhibitory effect of ADAMTS13 on iron deposition in renal tissue (Figure 3a). In addition, compared with CP+ADAMTS13 group, the expressions of total iron and Fe^2+^ in renal tissue were further decreased after the addition of Fer-1 (Figure 3b), accompanied by the increased expressions of SLC7A11, GPX4, FTH1, and FPN1. After Fe addition, the expressions of total iron and Fe^2+^ in renal tissues were significantly increased, accompanied by a significant decrease in the expressions of SLC7A11, GPX4, FTH1, and FPN1. Serum renal function indexes showed that compared with CP+ADAMTS13 group, the expressions of Scr and BUN were further decreased after the addition of Fer-1, while the expressions of Scr and BUN were significantly increased after the addition of Fe (Figure 4a). HE and PAS staining results showed that renal injury was significantly reduced after the addition of Fer-1, and further renal injury occurred after the addition of Fe (Figure 4b and c).The results of KIM1 and NGAL showed that the trend was consistent with the trend of pathological changes (Figure 4d). These results indicate that Fer-1 partially reverses CP induced AKI, while Fe aggravates this effect.

### rhADAMTS13 alleviated inflammatory response and oxidative stress in CP-induced AKI mice

**Figure 5.**
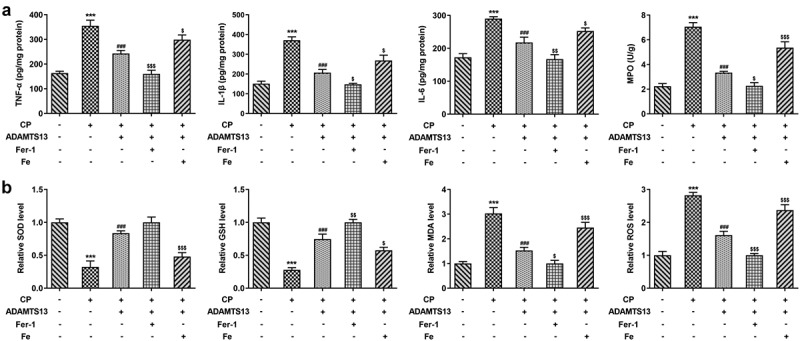
rhADAMTS13 alleviated inflammatory response and oxidative stress in CP-induced AKI mice. The levels of TNF-α, IL-1β, IL-6, MPO (a) and oxidative stress (b) in renal tissues were detected by ELISA. n = 5. ***P < 0.001 vs control. ###P < 0.001 vs CP. $p < 0.05, $$p < 0.01, $$$p < 0.001 vs CP + ADAMTS13

Next, we examined the inflammatory response and oxidative stress in the mice. ELISA results showed that the levels of TNF-α, IL-1β, IL-6 and MPO in renal tissue of mice induced by CP were significantly increased compared with control group. The expression of inflammatory cytokines was significantly decreased after the addition of ADAMTS13, and further decreased after the addition of Fer-1. However, the inhibitory effect of ADAMTS13 on inflammatory cytokines was reversed with the addition of Fe (Figure 5a). Compared with the control group, the levels of MDA and ROS were increased and the expressions of SOD and GSH were decreased after CP induction. After adding ADAMTS13, the level of MDA and ROS decreased and the expression of SOD and GSH increased. After adding Fer-1, the level of MDA and ROS decreased and the expression of SOD and GSH increased further. However, with the addition of Fe, the inhibitory effect of ADAMTS13 on oxidative stress was reversed (Figure 5b). The results showed that rhADAMTS13 reduced the inflammatory response and oxidative stress induced by CP in AKI mice.

### rhADAMTS13 regulated the Nrf2 signaling pathway

**Figure 6.**
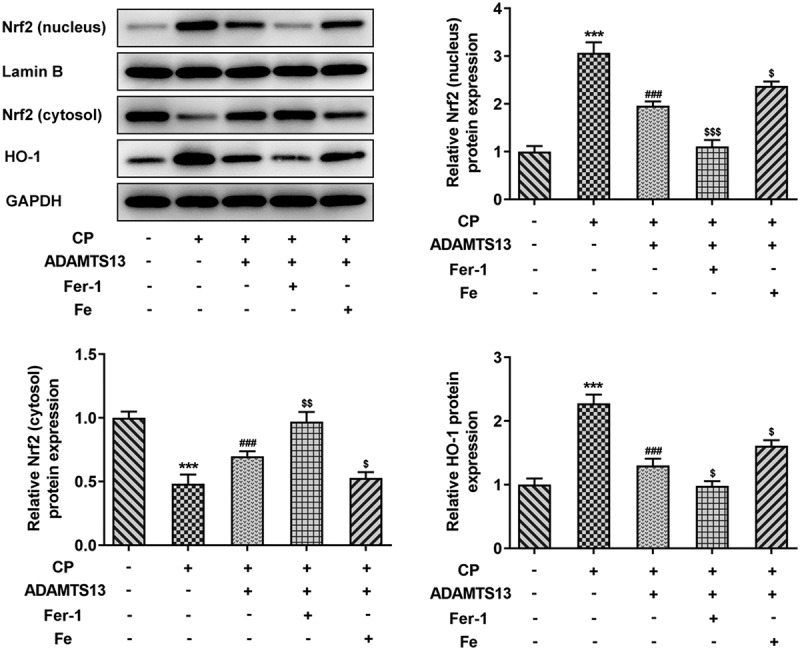
rhADAMTS13 regulated the NRF2 signaling pathway. Western blot detected the expression of nucleus Nrf2 and cytosol Nrf2 and HO-1. n = 3. ***P < 0.001 vs control. ###P < 0.001 vs CP. $p < 0.05, $$$p < 0.001 vs CP + ADAMTS13

We further discuss the mechanism and found that the Nrf signaling pathway was abnormal. Nrf2 nucleoprotein expression and downstream regulatory factor HO-1 expression were significantly increased in CP induced AKI. After rhADAMTS13 administration, the expression of Nrf nucleoprotein and HO-1 in renal tissue was decreased, while the expression of Nrf protein in cytoplasm was increased. Compared with CP+ADAMTS13, the expression of Nrf nucleoprotein and HO-1 was further decreased and the expression of Nrf protein in cytoplasm was further increased after the administration of ferroptosis inhibitor Fer-1. Expression of Nrf protein and HO-1 was reversed after administration of iron supplement Fe (Figure 6).

## Discussion

CP is a commonly used chemotherapy drug in clinic, and AKI is a serious complication of CP treatment. Toxic effects of CP can cause significant renal damage, resulting in decreased urine volume, serum creatinine, and increased urea nitrogen levels. Microscopic morphology of renal tissue showed increased inflammatory cells and even fibrosis [26]. In our experiment, it was found that after CP induction, the expressions of serum renal function indexes Scr and BUN were significantly increased in mice, and pathological studies showed that renal tissue was seriously damaged. The model of AKI was successfully induced.

Previous study has shown that appropriate regulation of ADAMTS13 function can improve renal microcirculation and vascular function in nephrectomy-induced renal injury disease, providing a new option for the treatment of AKI [27]. ADAMTS13 inhibited oxidative stress and ameliorated progressive chronic kidney disease after ischemia/reperfusion injury [15]. In our experiment, we found that administration of rhADAMTS13 significantly improved CP-induced AKI. This is consistent with literature.

Then we further discuss the mechanism. We found that iron was deposited in renal tissues of mice induced by CP, and the levels of total iron and Fe^2+^ were significantly increased, indicating ferroptosis occurred in renal tissues of mice induced by CP. Study has shown that deferriamine provides significant functional and histological protection in CP induced acute renal failure (ARF). In the proximal tubules, mice knocked out FTH1 had more severe kidney damage after CP treatment compared with the control group [28]. These results suggest that ferroptosis plays an important role in CP induced AKI. In addition, through literature review, we found that in children with β-thalassemia, ADAMTS13 level was positively correlated with platelet count and negatively correlated with serum ferritin [21]. Therefore, we hypothesized that ADAMTS13 plays a regulatory role in AKI by regulating the iron content in renal tissues of mice. Our results showed that administration of rhADAMTS13 significantly reduced CP induced ferroptosis. To further confirm our conclusion, we added Fer-1 and Fe to detect renal injury in mice. The results showed that Fer-1 further reversed CP induced AKI in addition to rh ADAMTS13 administration, while Fe aggravated CP induced AKI.

In this process, we also found abnormal Nrf2/HO-1 signaling pathway. Studies have shown that formononetin protects CP-induced AKI by activating the PPARα/Nrf2/HO-1/NQO1 pathway [29]. Vincetine alleviates CP induced AKI in rats by inhibiting the NF-κb pathway and activating the Nrf2/ARE pathway [30]. In the experiment, we found that the expressions of nuclear Nrf2 and HO-1 in kidney tissues were activated after CP induction.Nrf2 and HO-1 were significantly inhibited after administration of rh ADAMTS13. Study shows that Nrf2 inhibits ferroptosis and protects intestinal ischemia-reperfusion induced acute lung injury by regulating SLC7A11 and HO-1 [31]. The p62-Keap1-Nrf2 pathway plays a central role in the protection of hepatocellular carcinoma cells against ferroptosis through upregulation of multiple genes involved in iron and ROS metabolism [19]. In our experiment, it was found that the expressions of Nrf2 and HO-1 in the nucleus were further inhibited after further administration of Fer-1, while the expressions of Nrf2 and HO-1 were reversed after administration of Fe, suggesting that rhADAMTS13 regulates the Nrf2 signaling pathway to inhibit ferroptosis, thereby improving acute kidney injury induced by CP.

GPX4 and SLC7A11 were down-regulated in the occurrence of ferroptosis. Deletion of the SLC7A11 or GPX4 gene leads to lipid peroxidation and ferroptosis in some cells or tissues [32]. Intracellular iron homeostasis is mainly regulated post-transcriptional by iron-metabolism-related genes through the iron-response element-iron-regulatory protein system, such as ferritin (including heavy chain FTH1 and light chain FTL) [33]. Iron output is mediated by Ferroportin1 (FPN1, also known as SLC11A3), which releases Fe^2+^ extracellular, and then under the influence of correlation, the Fe^2+^ is then oxidized to Fe^3+^ [34]. In our experiment, it was found that the expressions of GPX4, SLC7A11, FTH1 and FPN1 in renal tissues were significantly decreased after CP induction, indicating ferroptosis in renal tissues at this time.GPX4, SLC7A11, FTH1, and FPN1 expressions were reversed after administration of rhADAMTS13, indicating that ferroptosis was inhibited.

Our article also has limitations. the expression of ADAMTS-13 in kidney in the absence of added rhADAMTS-13 and the expression of ADAMTS-13 after added rh ADAMTS-13 were not detected, which will be further discussed in the following experiment. In addition, we will further verify our experimental results in cell experiments in the following experiments.

## Conclusion

Our paper explores the mechanism of CP induced AKI and confirms that ADAMTS-13 regulates Nrf2 signaling pathway to inhibit ferroptosis, thereby ameliorating CP-induced acute kidney injury. This paper provides a theoretical basis for the treatment of AKI induced by CP.

## Data Availability

The datasets analyzed during the current study are available from the corresponding author on reasonable request.

## Research highlights

ADAMTS-13 improves CP-induced acute kidney injury

ADAMTS-13 down-regulates the expression of Nrf2 nuclear protein and HO-1

ADAMTS-13 inhibits ferroptosis by regulating Nrf2 signaling pathway
